# Supplementary material for: Assessment of cardiovascular risk in a slum population in Kenya: use of World Health Organisation/International Society of Hypertension (WHO/ISH) risk prediction charts - secondary analyses of a household survey
Source: BMJ Open. 2019 Sep 4;9(9):e029304. doi: 10.1136/bmjopen-2019-029304 (PMC6731939; doi:10.1136/bmjopen-2019-029304)
Supplement: Author's manuscript [file bmjopen-2019-029304.draft_revisions.pdf]

BMJ Open is committed to open peer review. As part of this commitment we make the peer review history of every article we publish publicly available.

When an article is published we post the peer reviewers' comments and the authors' responses online. We also post the versions of the paper that were used during peer review. These are the versions that the peer review comments apply to.

The versions of the paper that follow are the versions that were submitted during the peer review process. They are not the versions of record or the final published versions. They should not be cited or distributed as the published version of this manuscript.

BMJ Open is an open access journal and the full, final, typeset and author-corrected version of record of the manuscript is available on our site with no access controls, subscription charges or pay-per-view fees (http://bmjopen.bmj.com).

If you have any questions on BMJ Open's open peer review process please email
info.bmjopen@bmj.com

BMJ Open

Assessment of cardiovascular risk in a slum population in Kenya: use of World Health Organization/International Society of Hypertension (WHO/ISH)risk prediction charts

Journal:	*BMJ Open*
Manuscript ID	bmjopen-2019-029304
Article Type:	Research
Date Submitted by the Author:	22-Jan-2019
Complete List of Authors:	Vusirikala, Amoolya; University of Warwick Warwick Medical School,<br>Wekesah, Frederick; African Population and Health Research Center<br>Kyobutungi, Catherine; African Population and Health Research Center<br>Oyebode, Oyinlola; University of Warwick Warwick Medical School
Keywords:	Cardiac Epidemiology < CARDIOLOGY, PUBLIC HEALTH, Health policy < HEALTH SERVICES ADMINISTRATION & MANAGEMENT

SCHOLARONE™
Manuscripts

Assessment of cardiovascular risk in a slum population in Kenya: use of World Health Organization/International Society of Hypertension (WHO/ISH) risk prediction charts

Authors:

Vusirikala A*, MBChB

Wekesah M F, MSc

Kyobutungi C, PhD

Oyebode O, PhD

Affiliations:

AV; OO – University of Warwick, Coventry, CV4 7AL, United Kingdom

FW; CK – African Population Health Research Centre, APHRC Campus, Kitisuru, Nairobi, Kenya

FW; Julius Global Health, Julius Center for Health Sciences and Primary care, University Medical Center Utrecht, Utrecht University, the Netherlands

Corresponding Author and Address

Dr Amoolya Vusirikala

Warwick Medical School, University of Warwick, Coventry, CV4 7AL, United Kingdom

Telephone number: 07931608063

Email address: A.Vusirikala@warwick.ac.uk

Word Count: 2638

For peer review only

For peer review only - http://bmjopen.bmj.com/site/about/guidelines.xhtml

Abstract

Objectives: Although cardiovascular disease (CVD) is of growing importance in low- and middle-income countries (LMICs), there are conflicting views regarding CVD as a major public health problem for the urban poor, including those living in slums. We examine multivariable risk prediction in a slum population and assess the number of cardiovascular related deaths within 10 years of application of the tool.

Setting: We use data from the Nairobi Urban Health Demographic Surveillance population (residents of two slum communities) between May 2008 and April 2009.

Design: This is a secondary data analysis from a cross-sectional survey. We use the World Health Organisation/ International Society of Hypertension (WHO/ISH) cardiovascular risk prediction tool to examine 10-year risk of major CVD events in a slum population. CVD deaths in the cohort, reported up until June 2018 and identified through verbal autopsy are also presented.

Participants: 3063 men and women aged over 40 years with complete data for variables needed for the WHO/ISH risk prediction tool were eligible to take part.

Results: The majority of study members (2895, 94.5%) were predicted to have “low” risk (<10%) of a cardiovascular event over the next 10 years and just 51 (1.7%) to have “high” CVD risk (>=20%). 91 CVD deaths were reported for the cohort up until June 2018. Of individuals classified as low risk, 74 (2.6%) were identified as having died of CVD. Nine (7.7%) of individuals classified at 10-20% risk and eight (15.9%) classified at >20% were identified as dying of CVD.

Conclusions: This is a low risk population profile in comparison to results from application of multivariable risk prediction tools in other LMIC populations. This indicates that CVD may be lesser issue in slums than in other areas of LMICs cities. This has implications for health service planning in these contexts.

For peer review only - http://bmjopen.bmj.com/site/about/guidelines.xhtml

Strengths and limitations

- To the best of our knowledge this is the first study to apply a multivariable risk prediction tool to a population in a slum or informal settlement.
- We were able to identify CVD deaths of participants occurring in the slum during the 10 years after risk prediction.
- We were unable to exclude individuals with previous myocardial infarction as information was not available from the survey.
- Applying the risk score chart to cross-sectional population data may have underestimated the total CVD risk, as data that are required for thorough evaluation of total risk (such as family history) was absent.

peer review only

For peer review only - http://bmjopen.bmj.com/site/about/guidelines.xhtml

Introduction

Non-communicable diseases (NCDs) are the leading cause of death globally and have become the leading cause of deaths in low- and middle-income countries (LMICs) [1]. Cardiovascular disease (CVD) is a key player in this epidemic, accounting for most NCD deaths, and studies of CVD in urban areas of LMICs have suggested that risk is growing [2-4].

A large proportion of the world's urban population live in slums- neighbourhoods that are often informal, with poor housing and inadequate services [5]. There are conflicting views regarding CVD as a major public health problem for the urban poor, including those living in slums. An overview of health in slums found no synthesised evidence on CVD prevalence or the prevalence of CVD risk factors, while primary studies indicated that some CVD risk factors appear to be less prevalent among those living in slums than in their non-slum urban counterparts [5]. However, other primary studies carried out in urban LMICs have indicated that CVD risk is inversely associated with socio-economic status, or that there is no strong socio-economic gradient, which would suggest that those living in slums had at least equivalent or higher risk than other urban residents [6, 7].

Conventionally, CVD risk prediction focused on the presence of certain individual risk factors (e.g. elevated blood pressure or serum cholesterol), however the recognition of the multifactorial aetiology of CVD has led to a drastic shift away from the single risk factor approach toward a multivariable risk prediction approach. Taking into account the co-existence of multiple risk factors to determine CVD risk has been supported by much research that clearly demonstrates that the risk of a CVD event can differ among individuals with the same high levels of single risk factors due to the presence or absence of other risk factors [8-10]. Furthermore, studies have shown that identifying individuals at high CVD risk by adopting a total CVD risk assessment approach is more cost-effective method of CVD prevention especially in low resource settings [11, 12]. Determining total CVD risk requires risk prediction tools. The World Health Organisation/International Society of Hypertension (WHO/ISH) developed risk score prediction charts for different WHO subregions for the purposes of enabling clinicians to quickly assess total CVD risk in their patients, but also allows for risk stratification of a population in a simple manner.

However, there are no existing studies that have assessed multivariable risk prediction of CVD in a slum population. Therefore, the primary aim of this study is to apply the World Health Organisation/International Society of Hypertension (WHO/ISH) risk prediction tool to a slum population in Nairobi, Kenya. In addition, we were able to assess the number of cardiovascular related deaths occurring within the slum (but not non-fatal events, or fatal

For peer review only - http://bmjopen.bmj.com/site/about/guidelines.xhtml

events occurring elsewhere) reported within 10 years of application of the tool, giving us some idea about the utility of the WHO/ISH tool in this population, and about the burden of CVD within the slum setting. These findings will inform plans for health service delivery in the context of urban poor settings.

Methods

Study Population

This study utilizes data from a cross-sectional survey conducted by the African Population and Health Research Center (APHRC) within the Nairobi Urban Health Demographic Surveillance Site (NUHDSS) population between May 2008 and April 2009. The NUHDSS was set up to examine the long-term social, economic and health effects of two slum communities within the city of Nairobi, Korogocho and Viwandani [13]. This population-based survey utilized the sampling frame from the NUHDSS and a stratified, sampling strategy based on the WHO STEPwise protocol with a target of 250 respondents in each of the following strata: sex, age group (18-24, 25-30, 31-40, 41-50, 51-60, and 60+), and slum of residence (Korogocho and Viwandani). Data were collected from a total of 5,470 individuals aged 18 years and above. Further details on the sampling frame and data collection methods are published elsewhere [14]. Men and women aged over the age of 40 years with complete data for variables needed for the WHO/ISH risk prediction tool were eligible to take part in this secondary data analysis.

Study tool

In order to assess the 10- year risk of fatal & non-fatal cardiovascular disease (namely myocardial infarction or stroke) for each participant in our sample, we used the World Health Organisation/International Society of Hypertension (WHO/ISH) risk prediction charts for Africa sub-region (AFR E). The charts are designed for those over 40 and those who do not have established coronary heart disease (CHD), stroke or other atherosclerotic disease. Therefore our study sample excluded those <40 years of age and those with stroke. We were unable to identify and exclude those who had established CHD or other atherosclerotic disease as the information was not available from the survey.

The chart requires data on sex, age, systolic blood pressure, smoking status and presence or absence of diabetes mellitus. If total serum cholesterol is available, this is used in CVD risk prediction, however there is also an algorithm for use where no total serum cholesterol record is available, which we used to calculate risk for the study participants with missing

For peer review only - http://bmjopen.bmj.com/site/about/guidelines.xhtml

cholesterol data. Studies have demonstrated high correlation between laboratory-based scores and non-laboratory based scores for men and women [15, 16].

Following guidelines for using the WHO/ISH risk prediction tool variables were constructed as follows: Smokers were considered as those who were current smokers at assessment or those who quit smoking within the last year before the assessment; Presence of diabetes was defined as someone taking insulin or oral hypoglycaemic drugs or having a study-measured OGTT fasting glucose > 7.0 mmol/l; Systolic blood pressure was the average of three readings on day of survey while study member was seated using OMRON M6 blood pressure machine; Total cholesterol (mmol/l) was measured by taking capillary blood from fingertips using the ACCUCHECK GCT monitors and test strips. The predicted risk falls into categories including: from <10% (low), 10- <20% (intermediate), 20- <30%, 30- <40% and 40% or more (high).

In addition, practice points accompany the WHO risk prediction charts and state that CVD risk may be elevated over that specified by the charts when certain factors are present. We were able to obtain the following CVD enhancing risk factors for our study members: raised triglycerides (>2.0 mmol/l), whether on hypertensive medication and presence of obesity defined according to BMI (weight in kg divided by the square of height in cm). The prevalence of these CVD risk elevating factors were tabulated by the different risk categories.

Identifying cardiovascular related deaths within 10 years of application of the tool

To assess the cardiovascular related deaths among the participants of this study, verbal autopsy data present for all deaths recorded between 2008 and June 2018 was obtained from APHRC. A record linkage was undertaken between the cross-sectional survey and the verbal autopsy data using a unique identifier present in both data sources.

Verbal autopsy interviews are conducted by experienced field interviewers with a “credible respondent”, usually a family member following identification of deaths during regular Demographic Surveillance Site (DSS) data collection [17]. A standardised questionnaire developed in conjunction with other International network of Demographic Evaluation of Populations and Their Health (INDEPTH) sites is used and consists of open and closed questions focusing on events leading to the death and specific clinical signs and symptoms that the deceased had prior to their death. After several visits to a household, if no “credible respondent” is identified, verbal autopsy is coded as missing, and no cause of death is recorded.

For peer review only - http://bmjopen.bmj.com/site/about/guidelines.xhtml

For peer review only

Cause of death is then generated using InterVA-4 software, which uses probabilistic models based on Bayes' theorem to interpret symptom and signs data from verbal autopsy questionnaires and determine possible causes of death. Detailed information of the InterVA model and how it was developed have been described in previous studies [18-20]. Those with a cardiovascular code recorded under the variable first broad cause of death was used to define cardiovascular related death in this study. We additionally examined deaths reported due to diabetes mellitus because someone who had diabetes at the time of death may have died from a cardiovascular outcome, but credible family members may only have discussed the condition they were suffering prior to death.

Please note that the deaths recorded in this study were deaths only identified during the regular data collection rounds by the DSS team, it may be that more deaths occurred among participants of the original cross-sectional survey that were not identified (for example, the participant had moved before the death took place) and non-fatal CVD events were not captured at all. We were not able to link individuals in this dataset to know if they were still resident in the NUHDSS in June 2018- but a larger study sample, including this one, identified just 53/4290 (1.2%) had exited the NUHDSS between 2008 and 2018 (Frederick Wekesah- personal communication). The published rate of out migration between 2003 and 2012 was 22.5% [13]. For these reasons we can't be sure how well the tool predicted CVD events in this population but include these figures to add to knowledge about the burden of disease and give some indication of the tool's performance.

Patients and public were not involved in this study.

Statistical Analyses

Data analysis was conducted in STATA, version 14.0 (StataCorp. LP, College Station, USA). Percentages were calculated for categorical variables.

Results

Description of sample

Of the 5470 survey participants, 2,316 were excluded due to age (<40 years). Of the remaining 3,154 participants aged over 40, 91 (2.9%) were excluded due to incomplete data for the variables required of the WHO/ISH risk prediction tool.

Characteristics of the 3063 participants included in the final sample are represented in Table 1. The majority of participants included in the analyses were male (57.6%) and were between

For peer review only

For peer review only - http://bmjopen.bmj.com/site/about/guidelines.xhtml

40-60 years old, 88% of participants included in the analyses were non-smokers, 3% had diabetes and approximately 24% had blood pressure >=140 mmHg.

Table 1: Characteristics of study participants

	Total (n= 3063)	Men (n=1765)	Women (n=1298)
**Age in years (n, %)**			
40-49	1168 (38.13)	595 (33.71)	573 (44.14)
50-59	1169 (38.17)	770 (43.63)	399 (30.74)
60-69	493 (16.10)	294 (16.66)	199 (15.33)
>=70	233 (7.61)	106 (6.01)	127 (9.78)
**BMI (kg/m^2)**			
<30	2714	1700	1014
>=30	322	53	269
**Smoking status (n, %)**			
Current	375 (12.24)	364 (20.62)	11 (0.85)
Non-smoker	2688 (87.76)	1401(79.38)	1287 (99.15)
**Blood pressure (n, %)**			
<140	2312 (75.48)	1382 (78.30)	939 (72.34)
140-159	441 (14.40)	237 (13.43)	204 (15.72)
160-179	185 (6.04)	101 (5.72)	84 (6.47)
180+	116 (3.79)	45 (2.55)	71 (5.47)
**Diabetes (n, %)**			
Absent	2976 (97.16)	1727 (97.85)	1249 (96.22)
Present	87 (2.84)	38 (2.15)	49 (3.78)
**Total Cholesterol (n, %)**			
<5	2220 (72.48)	1307 (74.05)	913 (70.33)
5-5.9	516 (16.84)	281 (15.92)	235 (18.10)
6-6.9	74 (2.42)	32 (1.81)	42 (3.24)
7-7.9	10 (0.33)	5 (0.28)	5 (0.39)
8+	2 (0.07)	1 (0.06)	1 (0.08)
Cholesterol missing	241(7.87)	139 (7.88)	102 (7.86)

For peer review only - http://bmjopen.bmj.com/site/about/guidelines.xhtml

Table 2: Proportion of population % at different level of CVD risk & Prevalence of CVD risk enhancing factors

10-year CVD risk	<10%	10 to <20%	20% to < 30%	30% to <40%	>=40%
CVD risk-prediction from WHO/ISH chart alone (n, %)	2895 (94.5)	117 (3.8)	31 (1.0)	14 (0.5)	6 (0.2)
CVD enhancing factors					
Obesity (BMI >=30) (n, % of risk category)	292 (10.2)	21 (18.3)	9 (29.0)	0 (0)	0 (0)
On anti-hypertensive medication (n, % of risk category)	75 (2.6)	20 (17.1)	6 (19.4)	1 (7.1)	2 (33.3)
High Triglycerides (n, % risk category [a])	714 (27.6)	46 (42.6)	12 (44.4)	3 (25.0)	4 (80.0)

[a] High Triglycerides were recorded in 2739 participants of our total sample size of 3063

b Realistic BMI values only available for 3036 participants of our total sample size of 3063

The majority of participants in this study sample had low (<10%) total 10-year CVD risk (2895, 94.5%) [Table 2]. When CVD-risk enhancing factors were taken into account 1963 participants (64.1%) were low (<10%) total 10-year CVD risk with no additional risk enhancing factors.

WHO also states risk from prediction charts alone can underestimate the risk in those with high blood pressure (>=160/100) or blood cholesterol >=8. In our sample, 9.83% had raised blood pressure but only 0.07% of those who had a cholesterol blood test were raised above the specified level. Of those at <10% risk group, 131 participants had raised blood pressure above or equal to 160/100 and of those in this category that had a blood cholesterol test only 2 participants had a blood cholesterol >=8.

For peer review only - http://bmjopen.bmj.com/site/about/guidelines.xhtml

Table 3: Total Deaths and deaths due to cardiovascular disease recorded up to June 2018 at different levels of predicted CVD risk (based on chart alone)

**10-year CVD risk**	<10%	10-20%	20-30%	30-40%	>=40%
Total Deaths recorded (n, %)	336 (11.6)	25 (21.4)	11 (35.5)	7 (50.0)	1 (16.7)
Broad 1st cause of death: Cardiovascular (n, %)	74 (2.6)	9 (7.7)	5 (16.1)	2 (14.3)	1 (16.7)
Broad 1st cause of death: Diabetes Mellitus (n, %)	8 (0.3)	0 (0.0)	0 (0.0)	0 (0.0)	0 (0.0)
Indeterminate cause (n, %)	30 (1.0)	4 (3.4)	0 (0.0)	0 (0.0)	0 (0.0)
VA not done (n, %)	33 (1.1)	1 (0.9)	0 (0.0)	0 (0.0)	0 (0.0)

Following record linkage of verbal autopsy database and the respondents of the cross-sectional survey, 466 records were matched i.e.: 466 of the original cohort had a death reported up to June 2018 among the participants from the cross-sectional survey (n=5,470). Among the 3063 participants included in this study, 410 deaths were recorded with 91 deaths specifically related to CVD (3% of the study population), while 34 were classified as indeterminate and in 34 further cases, a verbal autopsy was not performed. Cardiovascular related cause of death was assigned to 74 (2.6%) of individuals classified as low risk (<10%), 9 (7.7%) of individuals classified at 10-20% risk, and 8 (15.9%) of those at high risk (>=20%) [Table 3].

Discussion

Of the 3063 study members aged over 40, the majority (94.5%) were predicted to have a less than 10% chance experiencing a cardiovascular event over the next 10 years, just 1.7% had a "high" CVD risk (>=20%). This is a low risk population profile in comparison to results from application of the multivariable risk prediction tools in other populations. Studies conducted specifically among urban dwellers in LMIC countries such as Malaysia [21] and Sri Lanka [22] have found 20.5% and 8.2% individuals were at high risk (>=20%) of having a future CVD event, respectively. Furthermore, the proportion of individuals in our study shown to be at low risk of a CVD event over 10 years (< 10% risk) was higher than that of studies who

For peer review only - http://bmjopen.bmj.com/site/about/guidelines.xhtml

used the WHO/ISH risk prediction charts carried out in rural Nepal (86.4%) [23], rural South India (83%) [24] and rural Bangladesh (81.3%) [25]. Mendis et al. reported total 10-year CVD risk in defined geographical areas of seven countries including both urban and rural populations, but only two countries had a higher percentage of individuals classified as low risk in comparison to our study (lower: Iran (93.9%), Cuba (89.7%), Nigeria (86.0%), Georgia (83.1%), Pakistan (79.2%); similar: China (96.1%) and Sri Lanka (94.9%)) [26]. However, it is important to note, the proportion of individuals estimated to have low (<10%) total CVD risk substantially decreased in our study, when risk-elevating factors stated in practice points accompanying WHO/ISH charts (raised triglycerides, obesity and anti-hypertensive medication) were added to the CVD risk assessment of the population.

CVD deaths occurring in our study population within the slum, reflected risk-categories assigned by the WHO/ISH tool (bearing in mind that additional deaths may have occurred in study members outside the slum and that non-fatal events were not recorded). Taken in the whole it appears from this data that health services geared towards CVD prevention and treatment may be less of a priority in slum settings than in the wider urban areas of LMIC cities.

Certain limitations of this work need to be considered when interpreting the findings. First, we were unable to exclude individuals with previous myocardial infarction as information was not available from the survey. However, if we failed to identify significant numbers with a previous MI, the remaining population (once these individuals had been excluded) would have likely had an even more extremely low risk profile for CVD. Second, applying the risk score chart to cross-sectional population data may have underestimated the total CVD risk, as data that are required for thorough evaluation of total risk such as family history or even history of relevant current diseases (the obvious example being myocardial infarction) and treatments, were not present in the data. Finally, it is a regret that we don't have data on all possible fatal CVD events (for example in those who have moved from the study site and are therefore not followed up in the NUHDSS) or non-fatal events that have occurred in the 10 year since the risk data was collected in order to validate the WHO/ISH tool in this setting.

Despite these limitations, our study uses rare data to provide a good estimate of total 10-year CVD risk among a marginalised population in an urban poor setting in Africa. To the best of our knowledge this is the first study to apply a multivariable risk prediction tool to a population in a slum or informal settlement and to assess the number of cardiovascular related deaths within 10 years of application of the tool. This study shows there is a low risk

For peer review only - http://bmjopen.bmj.com/site/about/guidelines.xhtml

profile of CVD in this slum population in Nairobi, Kenya. This has implications for planning of health service delivery in slums.

Conflict of Interest: None to declare

Funding: This research did not receive any specific grant from funding agencies in the public, commercial, or not-for-profit sectors.

For peer review only

REFERENCES

1 Alwan A, Armstrong T, Bettcher D, et al. Global status report on noncommunicable diseases 2010: description of the global burden of NCDs, their risk factors and determinants. *World Health Organization* 2011.

2 Yusuf S, Rangarajan S, Teo K, et al. Cardiovascular risk and events in 17 low-, middle-, and high-income countries. *N Engl J Med* 2014;371:818-27.

3 Gaziano TA, Bitton A, Anand S, et al. Growing epidemic of coronary heart disease in low- and middle-income countries. *Curr Probl Cardiol* 2010;35:72-115.

4 Teo KK, Dokainish H. The emerging epidemic of cardiovascular risk factors and atherosclerotic disease in developing countries. *Can J Cardiol* 2017;33:358-65.

5 Ezeh A, Oyebode O, Satterthwaite D, et al. The history, geography, and sociology of slums and the health problems of people who live in slums. *The lancet* 2017;389:547-58.

6 Marins VM, Almeida RM, Pereira RA, et al. The association between socioeconomic indicators and cardiovascular disease risk factors in Rio de Janeiro, Brazil. *J Biosoc Sci* 2007;39:221-9.

7 Vellakkal S, Subramanian S, Millett C, et al. Socioeconomic inequalities in non-communicable diseases prevalence in India: disparities between self-reported diagnoses and standardized measures. *PloS one* 2013;8:e68219.

8 Volpe M, Alderman MH, Furberg CD, et al. *Beyond hypertension: Toward guidelines for cardiovascular risk reduction* 2004.

9 Andersson OK, Almgren T, Persson B, et al. Survival in treated hypertension: follow up study after two decades. *BMJ* 1998;317:167-71.

10 Tocci G, Valenti V, Sciarretta S, et al. Multivariate risk assessment and risk score cards in hypertension. *Vasc Health Risk Manag* 2007;3:313-20.

11 Ndindjock R, Gedeon J, Mendis S, et al. Potential impact of single-risk-factor versus total risk management for the prevention of cardiovascular events in Seychelles. *Bull World Health Organ* 2011;89:286-95.

12 Mendis S, Lindholm LH, Anderson SG, et al. Total cardiovascular risk approach to improve efficiency of cardiovascular prevention in resource constrain settings. *J Clin Epidemiol* 2011;64:1451-62.

13 Beguy D, Elung'ata P, Mberu B, et al. Health & demographic surveillance system profile: the Nairobi urban health and demographic surveillance system (NUHDSS). *Int J Epidemiol* 2015;44:462-71.

14 Van de Vijver, Steven JM, Oti SO, Agyemang C, et al. Prevalence, awareness, treatment and control of hypertension among slum dwellers in Nairobi, Kenya. *J Hypertens* 2013;31:1018-24.

For peer review only - http://bmjopen.bmj.com/site/about/guidelines.xhtml

15 Pandya A, Weinstein MC, Gaziano TA. A comparative assessment of non-laboratory-based versus commonly used laboratory-based cardiovascular disease risk scores in the NHANES III population. *PloS one* 2011;6:e20416.

16 Gaziano TA, Young CR, Fitzmaurice G, et al. Laboratory-based versus non-laboratory-based method for assessment of cardiovascular disease risk: the NHANES I Follow-up Study cohort. *The Lancet* 2008;371:923-31.

17 Oti SO, Kyobutungi C. Verbal autopsy interpretation: a comparative analysis of the InterVA model versus physician review in determining causes of death in the Nairobi DSS. *Population health metrics* 2010;8:21.

18 Byass P, Huong DL, Van Minh H. A probabilistic approach to interpreting verbal autopsies: methodology and preliminary validation in Vietnam. *Scand J Public Health* 2003;31:32-7.

19 Oti SO, Kyobutungi C. Verbal autopsy interpretation: a comparative analysis of the InterVA model versus physician review in determining causes of death in the Nairobi DSS. *Population health metrics* 2010;8:21.

20 Byass P, Fottrell E, Huong DL, et al. Refining a probabilistic model for interpreting verbal autopsy data. *Scand J Public Health* 2006;34:26-31.

21 Su TT, Amiri M, Mohd Hairi F, et al. Prediction of cardiovascular disease risk among low-income urban dwellers in metropolitan Kuala Lumpur, Malaysia. *BioMed research international* 2015;2015.

22 Ranawaka U, Wijekoon C, Pathmeswaran A, et al. Risk estimates of cardiovascular diseases in a Sri Lankan community 2016.

23 Khanal MK, Ahmed MM, Moniruzzaman M, et al. Total cardiovascular risk for next 10 years among rural population of Nepal using WHO/ISH risk prediction chart. *BMC research notes* 2017;10:120.

24 Ghorpade AG, Shrivastava SR, Kar SS, et al. Estimation of the cardiovascular risk using World Health Organization/International Society of Hypertension (WHO/ISH) risk prediction charts in a rural population of South India. *International journal of health policy and management* 2015;4:531.

25 Ahmed M, Moniruzzaman M, Chowdhury S, et al. Cardiovascular Risk Assessment Among Urban Population of Bangladesh Using WHO/ISH Risk Prediction Chart. 2015;44:202-.

26 Mendis S, Lindholm LH, Anderson SG, et al. Total cardiovascular risk approach to improve efficiency of cardiovascular prevention in resource constrain settings. *J Clin Epidemiol* 2011;64:1451-62.

For peer review only

For peer review only - http://bmjopen.bmj.com/site/about/guidelines.xhtml

BMJ Open

Assessment of cardiovascular risk in a slum population in Kenya: use of World Health Organization/International Society of Hypertension (WHO/ISH)risk prediction charts- Secondary analyses of a Household Survey

Journal:	*BMJ Open*
Manuscript ID	bmjopen-2019-029304.R1
Article Type:	Research
Date Submitted by the Author:	22-Jun-2019

Complete List of Authors:	Vusirikala, Amoolya; University of Warwick Warwick Medical School,<br>Wekesah, Frederick; African Population and Health Research Center<br>Kyobutungi, Catherine; African Population and Health Research Center<br>Oyebode, Oyinlola; University of Warwick Warwick Medical School

**Primary Subject Heading**:	Public health
Secondary Subject Heading:	Cardiovascular medicine, Health policy
Keywords:	Cardiac Epidemiology < CARDIOLOGY, PUBLIC HEALTH, Health policy < HEALTH SERVICES ADMINISTRATION & MANAGEMENT

SCHOLARONE™
Manuscripts

Assessment of cardiovascular risk in a slum population in Kenya: use of World Health Organization/International Society of Hypertension (WHO/ISH) risk prediction charts - Secondary analyses of a Household Survey

Authors:

Vusirikala A*, MBChB

Wekesah M F, MSc

Kyobutungi C, PhD

Oyebode O, PhD

Affiliations:

AV; OO – University of Warwick, Coventry, CV4 7AL, United Kingdom

FW; CK – African Population Health Research Centre, APHRC Campus, Kitisuru, Nairobi, Kenya

FW; Julius Global Health, Julius Center for Health Sciences and Primary care, University Medical Center Utrecht, Utrecht University, the Netherlands

Corresponding Author and Address

Dr Amoolya Vusirikala

Warwick Medical School, University of Warwick, Coventry, CV4 7AL, United Kingdom

Telephone number: 07931608063

Email address: amoolyav@gmail.com

Word Count: 2970

For peer review only

Abstract

Objectives: Although cardiovascular disease (CVD) is of growing importance in low- and middle-income countries (LMICs), there are conflicting views regarding CVD as a major public health problem for the urban poor, including those living in slums. We examine multivariable risk prediction in a slum population and assess the number of cardiovascular related deaths within 10 years of application of the tool.

Setting: We use data from the Nairobi Urban Health Demographic Surveillance population (residents of two slum communities) between May 2008 and April 2009.

Design: This is a secondary data analysis from a cross-sectional survey. We use the World Health Organisation/ International Society of Hypertension (WHO/ISH) cardiovascular risk prediction tool to examine 10-year risk of major CVD events in a slum population. CVD deaths in the cohort, reported up until June 2018 and identified through verbal autopsy are also presented.

Participants: 3063 men and women aged over 40 years with complete data for variables needed for the WHO/ISH risk prediction tool were eligible to take part.

Results: The majority of study members (2895, 94.5%) were predicted to have “low” risk (<10%) of a cardiovascular event over the next 10 years and just 51 (1.7%) to have “high” CVD risk (>=20%). 91 CVD deaths were reported for the cohort up until June 2018. Of individuals classified as low risk, 74 (2.6%) were identified as having died of CVD. Nine (7.7%) of individuals classified at 10-20% risk and eight (15.9%) classified at >20% were identified as dying of CVD.

Conclusions: This is a low risk population profile in comparison to results from application of multivariable risk prediction tools in other LMIC populations. This indicates that CVD may be lesser issue in slums than in other areas of LMICs cities. This has implications for health service planning in these contexts.

For peer review only - http://bmjopen.bmj.com/site/about/guidelines.xhtml

Strengths and limitations

- To the best of our knowledge this is the first study to apply a multivariable risk prediction tool to a population in a slum or informal settlement.
- We were able to identify CVD deaths of participants occurring in the slum during the 10 years after risk prediction.
- We were unable to exclude individuals with previous myocardial infarction as information was not available from the survey.
- Applying the risk score chart to cross-sectional population data may have underestimated the total CVD risk, as data that are required for thorough evaluation of total risk (such as family history) was absent.

peer review only

For peer review only - http://bmjopen.bmj.com/site/about/guidelines.xhtml

Introduction

Non-communicable diseases (NCDs) are the leading cause of death globally and have become the leading cause of deaths in low- and middle-income countries (LMICs).[1] Cardiovascular disease (CVD) is a key player in this epidemic, accounting for most NCD deaths, and studies of CVD in urban areas of LMICs have suggested that risk is growing.[2-4]

A large proportion of the world's urban population live in slums- neighbourhoods that are often informal, with poor housing and inadequate services.[5] There are conflicting views regarding CVD as a major public health problem for the urban poor, including those living in slums. An overview of health in slums found no synthesised evidence on CVD prevalence or the prevalence of CVD risk factors, while primary studies indicated that some CVD risk factors appear to be less prevalent among those living in slums than in their non-slum urban counterparts.[5] However, other primary studies carried out in urban LMICs have indicated that CVD risk is inversely associated with socio-economic status, or that there is no strong socio-economic gradient, which would suggest that those living in slums had at least equivalent or higher risk than other urban residents.[6, 7]

Conventionally, CVD risk prediction focused on the presence of certain individual risk factors (e.g. elevated blood pressure or serum cholesterol), however the recognition of the multifactorial aetiology of CVD has led to a drastic shift away from the single risk factor approach toward a multivariable risk prediction approach. Taking into account the co-existence of multiple risk factors to determine CVD risk has been supported by much research that clearly demonstrates that the risk of a CVD event can differ among individuals with the same high levels of single risk factors due to the presence or absence of other risk factors.[8-10] Furthermore, studies have shown that identifying individuals at high CVD risk by adopting a total CVD risk assessment approach is more cost-effective method of CVD prevention especially in low resource settings.[11, 12] Determining total CVD risk requires risk prediction tools. The World Health Organisation/International Society of Hypertension (WHO/ISH) developed risk score prediction charts for different WHO subregions for the purposes of enabling clinicians to quickly assess total CVD risk in their patients, but also allows for risk stratification of a population in a simple manner.[13]

However, there are no existing studies that have assessed multivariable risk prediction of CVD in a slum population. Therefore, the primary aim of this study is to apply the World Health Organisation/International Society of Hypertension (WHO/ISH) risk prediction tool to a slum population in Nairobi, Kenya. In addition, we were able to assess the number of cardiovascular related deaths occurring within the slum (but not non-fatal events, or fatal events occurring elsewhere) reported within 10 years of application of the tool, giving us

For peer review only - http://bmjopen.bmj.com/site/about/guidelines.xhtml

some idea about the utility of the WHO/ISH tool in this population, and about the burden of CVD within the slum setting. These findings will inform plans for health service delivery in the context of urban poor settings.

Methods

Study Population

This study utilizes data from a cross-sectional survey conducted by the African Population and Health Research Center (APHRC) within the Nairobi Urban Health Demographic Surveillance Site (NUHDSS) population between May 2008 and April 2009. The NUHDSS was set up to examine the long-term social, economic and health effects of two slum communities within the city of Nairobi, Korogocho and Viwandani.[14] This population-based survey utilized the sampling frame from the NUHDSS and a stratified, sampling strategy based on the WHO STEPwise protocol with a target of 250 respondents in each of the following strata: sex, age group (18-24, 25-30, 31-40, 41-50, 51-60, and 60+), and slum of residence (Korogocho and Viwandani). Data were collected from a total of 5,470 individuals aged 18 years and above. Further details on the sampling frame and data collection methods are published elsewhere.[15] Men and women aged over the age of 40 years with complete data for variables needed for the WHO/ISH risk prediction tool were eligible to take part in this secondary data analysis.

Ethics

Operations of the NUHDSS are approved by the Government of Kenya, and the ethical responsibilities for its operations overseen by the Kenya Medical Research Institute (KEMRI). The CVD study was approved by the Scientific and Ethics Review unit of KEMRI (SERU NON-SSC 339). Participants were made aware that their participation in the study was voluntary, and individual informed consent was sought from all participants before their involvement in the study.

Study tool

In order to assess the 10- year risk of fatal & non-fatal cardiovascular disease (namely myocardial infarction or stroke) for each participant in our sample, we used the World Health Organisation/International Society of Hypertension (WHO/ISH) risk prediction charts for Africa sub-region (AFR E).[13] The charts are designed for those over 40 and those who do not have established coronary heart disease (CHD), stroke or other atherosclerotic disease. Therefore, our study sample excluded those <40 years of age and those with stroke. We

For peer review only - http://bmjopen.bmj.com/site/about/guidelines.xhtml

were unable to identify and exclude those who had established CHD or other atherosclerotic disease as the information was not available from the survey.

The chart requires data on sex, age, systolic blood pressure, smoking status and presence or absence of diabetes mellitus. If total serum cholesterol is available, this is used in CVD risk prediction, however there is also an algorithm for use where no total serum cholesterol record is available, which we used to calculate risk for the study participants with missing cholesterol data. Studies have demonstrated high correlation between laboratory-based scores and non-laboratory based scores for men and women.[16, 17]

Following guidelines for using the WHO/ISH risk prediction tool variables were constructed as follows: smokers were considered as those who were current smokers at assessment or those who quit smoking within the last year before the assessment; presence of diabetes was defined as someone taking insulin or oral hypoglycaemic drugs or having a study-measured fasting glucose > 7.0 mmol/l; systolic blood pressure was the average of three readings on the day of survey while study member was seated using OMRON M6 blood pressure machine; total cholesterol (mmol/l) was measured by taking capillary blood from fingertips using the ACCUCHECK GCT monitors and test strips. The predicted risk falls into categories including: from <10% (low), 10- <20% (moderate), 20- <30% (high), 30- <40% (very high) and 40% or more (highest).[18]

In addition, practice points accompany the WHO risk prediction charts and state that CVD risk may be elevated over that specified by the charts when certain factors are present. We were able to obtain the following CVD enhancing risk factors for our study members: raised triglycerides (>2.0 mmol/l), whether on hypertensive medication and presence of obesity defined according to BMI (weight in kg divided by the square of height in cm). The prevalence of these CVD risk elevating factors were tabulated by the different risk categories.

Identifying cardiovascular related deaths within 10 years of application of the tool

To assess the cardiovascular related deaths among the participants of this study, verbal autopsy data present for all deaths recorded between 2008 and June 2018 was obtained from APHRC. A record linkage was undertaken between the cross-sectional survey and the verbal autopsy data using a unique identifier present in both data sources.

Verbal autopsy interviews are conducted by experienced field interviewers with a “credible respondent”, usually a family member following identification of deaths during regular Demographic Surveillance Site (DSS) data collection.[19] A standardised questionnaire

For peer review only - http://bmjopen.bmj.com/site/about/guidelines.xhtml

developed in conjunction with other International network of Demographic Evaluation of Populations and Their Health (INDEPTH) sites is used and consists of open and closed questions focusing on events leading to the death and specific clinical signs and symptoms that the deceased had prior to their death. After several visits to a household, if no “credible respondent” is identified, verbal autopsy is coded as missing, and no cause of death is recorded.

Cause of death is then generated using InterVA-4 software, which uses probabilistic models based on Bayes' theorem to interpret symptom and signs data from verbal autopsy questionnaires and determine possible causes of death. Detailed information of the InterVA model and how it was developed have been described in previous studies.[20-22] Those with a cardiovascular code recorded under the variable first broad cause of death was used to define cardiovascular related death in this study. CV death included ischaemic heart disease, cerebrovascular disease, hypertensive diseases, pulmonary heart disease and diseases of pulmonary circulation, and diseases of arteries, arterioles and capillaries. We additionally examined deaths reported due to diabetes mellitus because someone who had diabetes at the time of death may have died from a cardiovascular outcome, but credible family members may only have discussed the condition they were suffering prior to death.

Please note that the deaths recorded in this study were deaths only identified during the regular data collection rounds by the DSS team, it may be that more deaths occurred among participants of the original cross-sectional survey that were not identified (for example, the participant had moved before the death took place) and non-fatal CVD events were not captured at all. We were not able to link individuals in this dataset to know if they were still resident in the NUHDSS in June 2018- but a larger study sample, including this one, identified just 53/4290 (1.2%) had exited the NUHDSS between 2008 and 2018 (Frederick Wekesah- personal communication). The published rate of out migration between 2003 and 2012 was 22.5%.[14] For these reasons we can’t be sure how well the tool predicted CVD events in this population but include these figures to add to knowledge about the burden of disease and give some indication of the tool’s performance.

Patients and public were not involved in this study.

Statistical Analyses

Data analysis was conducted in STATA, version 14.0 (StataCorp. LP, College Station, USA). Percentages were calculated for categorical variables. Sampling weights were applied where noted.

For peer review only - http://bmjopen.bmj.com/site/about/guidelines.xhtml

Results

Description of sample

Of the 5470 survey participants, 2,316 were excluded due to age (<40 years). Of the remaining 3,154 participants aged over 40, 10 were excluded due to having a stroke. 81 (2.69%) were excluded due to incomplete data for the variables required of the WHO/ISH risk prediction tool (missing data for smoking status (n=2) and for blood pressure (n=81)).

Characteristics of the 3063 participants included in the final sample are represented in Table 1. The majority of participants included in the analyses were male (57.6%) and were between 40-60 years old, 88% of participants included in the analyses were non-smokers, 3% had diabetes and approximately 24% had blood pressure >=140 mmHg.

Table 1: Characteristics of study participants

	Total (n= 3063)	Men (n=1765)	Women (n=1298)
**Age in years (n, %)**			
40-49	1168 (38.13)	595 (33.71)	573 (44.14)
50-59	1169 (38.17)	770 (43.63)	399 (30.74)
60-69	493 (16.10)	294 (16.66)	199 (15.33)
>=70	233 (7.61)	106 (6.01)	127 (9.78)
**BMI (kg/m^2)**			
<30	2714	1700	1014
>=30	322	53	269
**Smoking status (n, %)**			
Current	375 (12.24)	364 (20.62)	11 (0.85)
Non-smoker	2688 (87.76)	1401(79.38)	1287 (99.15)
**Blood pressure (n, %)**			
<140	2312 (75.48)	1382 (78.30)	939 (72.34)
140-159	441 (14.40)	237 (13.43)	204 (15.72)
160-179	185 (6.04)	101 (5.72)	84 (6.47)
180+	116 (3.79)	45 (2.55)	71 (5.47)
**Diabetes (n, %)**			
Absent	2976 (97.16)	1727 (97.85)	1249 (96.22)
Present	87 (2.84)	38 (2.15)	49 (3.78)
**Total Cholesterol (n, %)**			
<5	2220 (72.48)	1307 (74.05)	913 (70.33)

For peer review only - http://bmjopen.bmj.com/site/about/guidelines.xhtml

5-5.9	516 (16.84)	281 (15.92)	235 (18.10)
6-6.9	74 (2.42)	32 (1.81)	42 (3.24)
7-7.9	10 (0.33)	5 (0.28)	5 (0.39)
8+	2 (0.07)	1 (0.06)	1 (0.08)
Cholesterol missing	241(7.87)	139 (7.88)	102 (7.86)

Table 2: Proportion of population % at different level of CVD risk & Prevalence of CVD risk enhancing factors

10-year CVD risk	<10% Low	10 to <20% Moderate	20% to < 30% High risk	30% to <40% Very High risk	>=40% Highest risk
**Total**					
CVD risk-prediction from WHO/ISH chart alone (n, %)	2895 (94.5)	117 (3.8)	31 (1.0)	14 (0.5)	6 (0.2)
CVD risk-prediction from WHO/ISH chart alone (%) (weighted)	(96.3)	(2.5)	(0.8)	(0.3)	(0.1)
One or more CVD enhancing factors (n, % of risk category)	932 (32.2)	62 (53.0)	20 (64.5)	3 (21.4)	5 (83.3)
Obesity (BMI >=30) (n, % of risk category [a])	292 (10.2)	21 (18.3)	9 (29.0)	0 (0)	0 (0)
On anti-hypertensive medication (n, % of risk category)	75 (2.6)	20 (17.1)	6 (19.4)	1 (7.1)	2 (33.3)
High Triglycerides (n, % risk category [b])	714 (27.6)	46 (42.6)	12 (44.4)	3 (25.0)	4 (80.0)
**Male**					
CVD risk-prediction from WHO/ISH chart alone (n, %)	1679 (95.1)	59 (3.3)	12 (0.7)	11 (0.6)	4 (0.2)
CVD risk-prediction from WHO/ISH chart alone (%) (weighted)	(95.2)	(3.5)	(1.1)	(0.2)	(0.1)
One or more CVD enhancing factors (n, % of risk category)	443 (26.4)	21 (35.6)	6 (50.0)	2 (18.1)	3 (75.0)

For peer review only

For peer review only - http://bmjopen.bmj.com/site/about/guidelines.xhtml

Obesity (BMI >=30) (n, % of risk category [a])	49 (2.9)	2 (3.4)	2 (16.7)	0 (0.0)	0 (0.0)
On anti-hypertensive medication (n, % of risk category)	22 (1.3)	6 (10.2)	1 (8.3)	0 (0.0)	0 (0.0)
High Triglycerides (n, % risk category [b])	400 (26.6)	16 (30.2)	4 (44.4)	2 (22.2)	3 (75.0)
**Female**					
CVD risk-prediction from WHO/ISH chart alone (n, %)	1216 (93.7)	58 (4.5)	19 (1.5)	3 (0.2)	2 (0.2)
CVD risk-prediction from WHO/ISH chart alone (%) (weighted)	(96.8)	(2.1)	(0.7)	(0.3)	(0.2)
One or more CVD enhancing factors (n, % of risk category)	489 (40.2)	41 (70.7)	14 (73.7)	1 (33.3)	2 (100)
Obesity (BMI >=30) (n, % of risk category [a])	243 (20.2)	19 (33.9)	7 (36.8)	0 (0.0)	0 (0.0)
On anti-hypertensive medication (n, % of risk category)	53 (4.4)	14 (24.1)	5 (26.3)	1 (33.3)	2 (100)
High Triglycerides (n, % risk category [b])	314 (28.9)	30 (54.5)	8 (44.4)	1 (33.3)	1 (100)

[a] *Realistic BMI values only available for 3036 participants of our total sample size of 3063*

[b] *High Triglycerides were recorded in 2739 participants of our total sample size of 3063*

The majority of participants in this study sample had low (<10%) total 10-year CVD risk (2895, 94.5%) [Table 2]. That is they had less than 10% predicted chance of a fatal or non-fatal CVD event over the following 10 years. When CVD-risk enhancing factors were taken into account 1963 participants (64.1%) were low (<10%) total 10-year CVD risk with no additional risk enhancing factors. In the weighted analysis, the percentage of people in the <10% risk group was 96.3% with the reduced risk profile due to correction of the over-sampling of older age groups. After applying the CVD-enhancing factors, the percentage of people in the <10% risk group reduced to 63.7% in the weighted analysis.

WHO also states risk from prediction charts alone can underestimate the risk in those with high blood pressure (>=160/100) or blood cholesterol >=8. In our sample, 9.83% had raised

For peer review only - http://bmjopen.bmj.com/site/about/guidelines.xhtml

blood pressure but only 0.07% of those who had a cholesterol blood test were raised above the specified level. Of those at <10% risk group, 131 participants had raised blood pressure above or equal to 160/100 and of those in this category that had a blood cholesterol test only 2 participants had a blood cholesterol >=8.

Table 3: Total Deaths and deaths due to cardiovascular disease recorded up to June 2018 at different levels of predicted CVD risk (based on chart alone)

10-year CVD risk	<10% Low	10-20% Moderate	20-30% High risk	30-40% Very High risk	>=40% Highest risk
**Total**					
Deaths recorded (n, %)	336 (11.6)	25 (21.4)	11 (35.5)	7 (50.0)	1 (16.7)
Broad 1st cause of death: Cardiovascular (n, %)	74 (2.6)	9 (7.7)	5 (16.1)	2 (14.3)	1 (16.7)
Broad 1st cause of death: Diabetes Mellitus (n, %)	8 (0.3)	0 (0.0)	0 (0.0)	0 (0.0)	0 (0.0)
Indeterminate cause (n, %)	30 (1.0)	4 (3.4)	0 (0.0)	0 (0.0)	0 (0.0)
VA not done (n, %)	33 (1.1)	1 (0.9)	0 (0.0)	0 (0.0)	0 (0.0)
**Male**					
Deaths recorded (n, %)	199 (11.9)	10 (16.9)	5 (41.7)	4 (36.4)	0 (0.0)
Broad 1st cause of death: Cardiovascular (n, %)	35 (2.1)	3 (5.1)	2 (16.7)	1 (9.1)	0 (0.0)
Broad 1st cause of death: Diabetes Mellitus (n, %)	3 (0.2)	0 (0.0)	0 (0.0)	0 (0.0)	0 (0.0)
Indeterminate cause (n, %)	16 (1.0)	1 (1.7)	0 (0.0)	0 (0.0)	0 (0.0)
VA not done (n, %)	22 (1.3)	1 (1.7)	0 (0.0)	0 (0.0)	0 (0.0)

For peer review only - http://bmjopen.bmj.com/site/about/guidelines.xhtml

**Female**					
Deaths recorded (n, %)	137 (11.3)	15 (25.9)	6 (31.6)	3 (100)	1 (50.0)
Broad 1st cause of death: Cardiovascular (n, %)	36 (3.1)	6 (10.3)	3 (15.8)	1 (33.3)	1 (50.0)
Broad 1st cause of death: Diabetes Mellitus (n, %)	5 (0.4)	0 (0.0)	0 (0.0)	0 (0.0)	0 (0.0)
Indeterminate cause (n, %)	141 (11.6)	3 (5.2)	0 (0.0)	0 (0.0)	0 (0.0)
VA not done (n, %)	11 (0.9)	0 (0.0)	0 (0.0)	0 (0.0)	0 (0.0)

Following record linkage of verbal autopsy database and the respondents of the cross-sectional survey, 466 records were matched i.e.: 466 of the original cohort had a death reported up to June 2018 among the participants from the cross-sectional survey (n=5,470). Among the 3063 participants included in this study, 410 deaths were recorded with 91 deaths specifically related to CVD (3% of the study population), while 34 were classified as indeterminate and in 34 further cases, a verbal autopsy was not performed. Cardiovascular related cause of death was assigned to 74 (2.6%) of individuals classified as low risk (<10% predicted chance of a fatal or non-fatal CVD event). Nine (7.7%) of individuals classified at 10-20% risk of a fatal or non-fatal CVD event were determined to have died from CVD, and 8 (15.9%) of those at high risk (>=20%) [Table 3]. Out of 336 deaths in <10% group, 87 individuals who had died had one or more CVD-enhancing factors, 18 of these deaths were due to CVD risk and two deaths were due to diabetes.

For peer review only

Discussion

Of the 3063 study members aged over 40, the majority (94.5%) were predicted to have a less than 10% chance experiencing a cardiovascular event over the next 10 years, just 1.7% had a “high” CVD risk (>=20%). When weighted to be representative of all adults aged over 40 living in the slum 96.3% were predicted to fall in the lowest risk group and just 1.2% have a “high” CVD risk (>=20%). This is a low risk population profile in comparison to results from application of the multivariable risk prediction tools in other populations. Studies conducted

For peer review only - http://bmjopen.bmj.com/site/about/guidelines.xhtml

specifically among urban dwellers in LMIC countries such as Malaysia [23] and Sri Lanka [24] have found 20.5% and 8.2% individuals were at high risk (>=20%) of having a future CVD event, respectively. Furthermore, the proportion of individuals in our study shown to be at low risk of a CVD event over 10 years (< 10% risk) was higher than that of studies who used the WHO/ISH risk prediction charts carried out in rural Nepal (86.4%),[25] rural South India (83%) [26] and rural Bangladesh (81.3%).[27] Mendis et al. reported total 10-year CVD risk in defined geographical areas of seven countries including both urban and rural populations, but only two countries had a higher percentage of individuals classified as low risk in comparison to our study (lower: Iran (93.9%), Cuba (89.7%), Nigeria (86.0%), Georgia (83.1%), Pakistan (79.2%); similar: China (96.1%) and Sri Lanka (94.9%)).[28] However, it is important to note, the proportion of individuals estimated to have low (<10%) total CVD risk substantially decreased in our study, when risk-elevating factors stated in practice points accompanying WHO/ISH charts (raised triglycerides, obesity and anti-hypertensive medication) were added to the CVD risk assessment of the population.

CVD deaths occurring in our study population within the slum, reflected risk-categories assigned by the WHO/ISH tool (bearing in mind that additional deaths may have occurred in study members outside the slum and that non-fatal events were not recorded). Taken in the whole it appears from this data that health services geared towards CVD treatment may be less of a priority in slum settings in Kenya, or potentially in sub-Saharan Africa, than in the wider urban areas of LMIC cities. An important reason for this may be the age-structure of the slum population, which is very young. However, given the large percentage of CVD-enhancing factors in this population, it could be that the future burden (when this population gets older) will be significant. The signal here could be that CVD prevention is more of a priority here than treatment.

Certain limitations of this work need to be considered when interpreting the findings. First, we were unable to exclude individuals with previous myocardial infarction as information was not available from the survey. However, if we failed to identify significant numbers with a previous MI, the remaining population (once these individuals had been excluded) would have likely had an even more extremely low risk profile for CVD. Second, applying the risk score chart to cross-sectional population data may have underestimated the total CVD risk, as data that are required for thorough evaluation of total risk such as family history or even history of relevant current diseases (the obvious example being myocardial infarction) and treatments, were not present in the data. Thirdly, there are some deviations in our methods from the instructions of how the WHO/ISH charts should be used: systolic blood pressure was measured three times on one day, rather than twice at two different time points, which

For peer review only - http://bmjopen.bmj.com/site/about/guidelines.xhtml

could increase the risk that some of the participants experienced white-coat hypertension; cholesterol (although optional) should be measured at two time points, we defined someone as have diabetes if they were taking insulin or oral hypoglycaemic drugs or if their fasting plasma glucose concentration was about 7.0mmol/l on one occasion (not on two separate occasions as recommended). Finally, where we used cholesterol readings- these were also from one time point, rather than two as recommended.

Finally, it is a regret that we don't have data on all possible fatal CVD events (for example in those who have moved from the study site and are therefore not followed up in the NUHDSS) or non-fatal events that have occurred in the 10 year since the risk data was collected in order to validate the WHO/ISH tool in this setting.

Despite these limitations, our study uses rare data to provide a good estimate of total 10-year CVD risk among a marginalised population in an urban poor setting in sub-Saharan Africa. To the best of our knowledge this is the first study to apply a multivariable risk prediction tool to a population in a slum or informal settlement and to assess the number of cardiovascular related deaths within 10 years of application of the tool. This study shows there is a low risk profile of CVD in this slum population in Nairobi, Kenya and that the WHO/ISH tool does differentiate groups at increasing risk of CVD mortality. This has implications for planning of health service delivery in slums.

Conflict of Interest: None to declare

Funding: OO and CK are supported by the National Institute for Health Research (NIHR) Research Unit on Improving Health in Slums. The original data on which the study is based was collected as part of research funded by the Wellcome Trust UK- Grant Number WT092775MA. This research did not receive any specific grant from funding agencies in the public, commercial, or not-for-profit sectors. This paper presented independent research and the views expressed are those of the authors and not necessarily those of the NHS, the HIGR or the Department of Health.

Contributors: OO conceived the study idea. OO, AV and FK contributed to the analysis plan. AV conducted the analyses. FK and CK provided advice on using the data. AV and OO wrote the first draft of the manuscript. All authors contributed to the final manuscript.

Data sharing statement: No additional data available

For peer review only - http://bmjopen.bmj.com/site/about/guidelines.xhtml

For peer review only

REFERENCES

1 Alwan A, Armstrong T, Bettcher D, et al. Global status report on noncommunicable diseases 2010: description of the global burden of NCDs, their risk factors and determinants. *World Health Organization* 2011.

2 Yusuf S, Rangarajan S, Teo K, et al. Cardiovascular risk and events in 17 low-, middle-, and high-income countries. *N Engl J Med* 2014;371:818-27.

3 Gaziano TA, Bitton A, Anand S, et al. Growing epidemic of coronary heart disease in low- and middle-income countries. *Curr Probl Cardiol* 2010;35:72-115.

4 Teo KK, Dokainish H. The emerging epidemic of cardiovascular risk factors and atherosclerotic disease in developing countries. *Can J Cardiol* 2017;33:358-65.

5 Ezeh A, Oyebode O, Satterthwaite D, et al. The history, geography, and sociology of slums and the health problems of people who live in slums. *The lancet* 2017;389:547-58.

6 Marins VM, Almeida RM, Pereira RA, et al. The association between socioeconomic indicators and cardiovascular disease risk factors in Rio de Janeiro, Brazil. *J Biosoc Sci* 2007;39:221-9.

7 Vellakkal S, Subramanian S, Millett C, et al. Socioeconomic inequalities in non-communicable diseases prevalence in India: disparities between self-reported diagnoses and standardized measures. *PloS one* 2013;8:e68219.

8 Volpe M, Alderman MH, Furberg CD, et al. *Beyond hypertension: Toward guidelines for cardiovascular risk reduction* 2004.

9 Andersson OK, Almgren T, Persson B, et al. Survival in treated hypertension: follow up study after two decades. *BMJ* 1998;317:167-71.

10 Tocci G, Valenti V, Sciarretta S, et al. Multivariate risk assessment and risk score cards in hypertension. *Vasc Health Risk Manag* 2007;3:313-20.

11 Ndindjock R, Gedeon J, Mendis S, et al. Potential impact of single-risk-factor versus total risk management for the prevention of cardiovascular events in Seychelles. *Bull World Health Organ* 2011;89:286-95.

12 Mendis S, Lindholm LH, Anderson SG, et al. Total cardiovascular risk approach to improve efficiency of cardiovascular prevention in resource constrain settings. *J Clin Epidemiol* 2011;64:1451-62.

For peer review only - http://bmjopen.bmj.com/site/about/guidelines.xhtml

13 . WHO/ISH Risk prediction charts for 14 WHO epidemiological sub-regions. 2007. Available from: http://ish-world.com/downloads/activities/colour_charts_24_Aug_07.pdf (accessed 18 Jun 2019).

14 Beguy D, Elung'ata P, Mberu B, et al. Health & demographic surveillance system profile: the Nairobi urban health and demographic surveillance system (NUHDSS). *Int J Epidemiol* 2015;44:462-71.

15 Van de Vijver, Steven JM, Oti SO, Agyemang C, et al. Prevalence, awareness, treatment and control of hypertension among slum dwellers in Nairobi, Kenya. *J Hypertens* 2013;31:1018-24.

16 Pandya A, Weinstein MC, Gaziano TA. A comparative assessment of non-laboratory-based versus commonly used laboratory-based cardiovascular disease risk scores in the NHANES III population. *PloS one* 2011;6:e20416.

17 Gaziano TA, Young CR, Fitzmaurice G, et al. Laboratory-based versus non-laboratory-based method for assessment of cardiovascular disease risk: the NHANES I Follow-up Study cohort. *The Lancet* 2008;371:923-31.

18 Organisation mondiale de la santé. Prevention of cardiovascular disease: guidelines for assessment and management of cardiovascular risk: World Health Organization 2007.

19 Oti SO, Kyobutungi C. Verbal autopsy interpretation: a comparative analysis of the InterVA model versus physician review in determining causes of death in the Nairobi DSS. *Population health metrics* 2010;8:21.

20 Byass P, Huong DL, Van Minh H. A probabilistic approach to interpreting verbal autopsies: methodology and preliminary validation in Vietnam. *Scand J Public Health* 2003;31:32-7.

21 Oti SO, Kyobutungi C. Verbal autopsy interpretation: a comparative analysis of the InterVA model versus physician review in determining causes of death in the Nairobi DSS. *Population health metrics* 2010;8:21.

22 Byass P, Fottrell E, Huong DL, et al. Refining a probabilistic model for interpreting verbal autopsy data. *Scand J Public Health* 2006;34:26-31.

23 Su TT, Amiri M, Mohd Hairi F, et al. Prediction of cardiovascular disease risk among low-income urban dwellers in metropolitan Kuala Lumpur, Malaysia. *BioMed research international* 2015;2015.

24 Ranawaka U, Wijekoon C, Pathmeswaran A, et al. Risk estimates of cardiovascular diseases in a Sri Lankan community. *Ceylon Med J.* 2016;61(1):11-7

25 Khanal MK, Ahmed MM, Moniruzzaman M, et al. Total cardiovascular risk for next 10 years among rural population of Nepal using WHO/ISH risk prediction chart. *BMC research notes* 2017;10:120.

26 Ghorpade AG, Shrivastava SR, Kar SS, et al. Estimation of the cardiovascular risk using World Health Organization/International Society of Hypertension (WHO/ISH) risk prediction charts in a rural population of South India. *International journal of health policy and management* 2015;4:531.

For peer review only - http://bmjopen.bmj.com/site/about/guidelines.xhtml

27 Ahmed M, Moniruzzaman M, Chowdhury S, et al. Cardiovascular Risk Assessment Among Urban Population of Bangladesh Using WHO/ISH Risk Prediction Chart. *Int J Epidemiol* 2015;44:i202.

28 Mendis S, Lindholm LH, Anderson SG, et al. Total cardiovascular risk approach to improve efficiency of cardiovascular prevention in resource constrain settings. *J Clin Epidemiol* 2011;64:1451-62.

For peer review only

STROBE Statement—Checklist of items that should be included in reports of ***cross-sectional studies***

	Item No	Recommendation	Reported on Page no.
**Title and abstract**	1	(*a*) Indicate the study's design with a commonly used term in the title or the abstract	1
		(*b*) Provide in the abstract an informative and balanced summary of what was done and what was found	2
**Introduction**			
Background/rationale	2	Explain the scientific background and rationale for the investigation being reported	4
Objectives	3	State specific objectives, including any prespecified hypotheses	4
**Methods**			
Study design	4	Present key elements of study design early in the paper	5-7
Setting	5	Describe the setting, locations, and relevant dates, including periods of recruitment, exposure, follow-up, and data collection	5-7
Participants	6	(*a*) Give the eligibility criteria, and the sources and methods of selection of participants	5
Variables	7	Clearly define all outcomes, exposures, predictors, potential confounders, and effect modifiers. Give diagnostic criteria, if applicable	5-7
Data sources/ measurement	8*	For each variable of interest, give sources of data and details of methods of assessment (measurement). Describe comparability of assessment methods if there is more than one group	5-7
Bias	9	Describe any efforts to address potential sources of bias	N/A
Study size	10	Explain how the study size was arrived at	8
Quantitative variables	11	Explain how quantitative variables were handled in the analyses. If applicable, describe which groupings were chosen and why	6-7
Statistical methods	12	(*a*) Describe all statistical methods, including those used to control for confounding	7
		(*b*) Describe any methods used to examine subgroups and interactions	N/A
		(*c*) Explain how missing data were addressed	6
		(*d*) If applicable, describe analytical methods taking account of sampling strategy	5, 7
		(*e*) Describe any sensitivity analyses	N/A
**Results**			
Participants	13*	(a) Report numbers of individuals at each stage of study—eg numbers potentially eligible, examined for eligibility, confirmed eligible, included in the study, completing follow-up, and analysed	8
		(b) Give reasons for non-participation at each stage	8
		(c) Consider use of a flow diagram	N/A
Descriptive data	14*	(a) Give characteristics of study participants (eg demographic, clinical, social) and information on exposures and potential confounders	8-9
		(b) Indicate number of participants with missing data for each variable of interest	8-10
Outcome data	15*	Report numbers of outcome events or summary measures	9-12
Main results	16	(*a*) Give unadjusted estimates and, if applicable, confounder-adjusted estimates and their precision (eg, 95% confidence interval). Make clear	N/A

For peer review only

		which confounders were adjusted for and why they were included	
		(*b*) Report category boundaries when continuous variables were categorized	N/A
		(*c*) If relevant, consider translating estimates of relative risk into absolute risk for a meaningful time period	N/A
Other analyses	17	Report other analyses done—eg analyses of subgroups and interactions, and sensitivity analyses	N/A
**Discussion**			
Key results	18	Summarise key results with reference to study objectives	12-13
Limitations	19	Discuss limitations of the study, taking into account sources of potential bias or imprecision. Discuss both direction and magnitude of any potential bias	13-14
Interpretation	20	Give a cautious overall interpretation of results considering objectives, limitations, multiplicity of analyses, results from similar studies, and other relevant evidence	14
Generalisability	21	Discuss the generalisability (external validity) of the study results	14
**Other information**			
Funding	22	Give the source of funding and the role of the funders for the present study and, if applicable, for the original study on which the present article is based	14

*Give information separately for exposed and unexposed groups.

Note: An Explanation and Elaboration article discusses each checklist item and gives methodological background and published examples of transparent reporting. The STROBE checklist is best used in conjunction with this article (freely available on the Web sites of PLoS Medicine at http://www.plosmedicine.org/, Annals of Internal Medicine at http://www.annals.org/, and Epidemiology at http://www.epidem.com/). Information on the STROBE Initiative is available at www.strobe-statement.org.

For peer review only

BMJ Open

Assessment of cardiovascular risk in a slum population in Kenya: use of World Health Organization/International Society of Hypertension (WHO/ISH)risk prediction charts- Secondary analyses of a Household Survey

Journal:	*BMJ Open*
Manuscript ID	bmjopen-2019-029304.R2
Article Type:	Research
Date Submitted by the Author:	09-Jul-2019
Complete List of Authors:	Vusirikala, Amoolya; University of Warwick Warwick Medical School,<br>Wekesah, Frederick; African Population and Health Research Center<br>Kyobutungi, Catherine; African Population and Health Research Center<br>Oyebode, Oyinlola; University of Warwick Warwick Medical School
**Primary Subject Heading**:	Public health
Secondary Subject Heading:	Cardiovascular medicine, Health policy
Keywords:	Cardiac Epidemiology < CARDIOLOGY, PUBLIC HEALTH, Health policy < HEALTH SERVICES ADMINISTRATION & MANAGEMENT

SCHOLARONE™
Manuscripts

Assessment of cardiovascular risk in a slum population in Kenya: use of World Health Organization/International Society of Hypertension (WHO/ISH) risk prediction charts - Secondary analyses of a Household Survey

Authors:

Vusirikala A*, MBChB

Wekesah M F, MSc

Kyobutungi C, PhD

Oyebode O, PhD

Affiliations:

AV; OO – University of Warwick, Coventry, CV4 7AL, United Kingdom

FW; CK – African Population Health Research Centre, APHRC Campus, Kitisuru, Nairobi, Kenya

FW; Julius Global Health, Julius Center for Health Sciences and Primary care, University Medical Center Utrecht, Utrecht University, the Netherlands

Corresponding Author and Address

Dr Amoolya Vusirikala

Warwick Medical School, University of Warwick, Coventry, CV4 7AL, United Kingdom

Telephone number: 07931608063

Email address: amoolyav@gmail.com

Word Count: 2957

For peer review only

Abstract

Objectives: Although cardiovascular disease (CVD) is of growing importance in low- and middle-income countries (LMICs), there are conflicting views regarding CVD as a major public health problem for the urban poor, including those living in slums. We examine multivariable risk prediction in a slum population and assess the number of cardiovascular related deaths within 10 years of application of the tool.

Setting: We use data from the Nairobi Urban Health Demographic Surveillance population (residents of two slum communities) between May 2008 and April 2009.

Design: This is a secondary data analysis from a cross-sectional survey. We use the World Health Organisation/International Society of Hypertension (WHO/ISH) cardiovascular risk prediction tool to examine 10-year risk of major CVD events in a slum population. CVD deaths in the cohort, reported up until June 2018 and identified through verbal autopsy are also presented.

Participants: 3063 men and women aged over 40 years with complete data for variables needed for the WHO/ISH risk prediction tool were eligible to take part.

Results: The majority of study members (2895, 94.5%) were predicted to have "low" risk (<10%) of a cardiovascular event over the next 10 years and just 51 (1.7%) to have "high" CVD risk (≥20%). 91 CVD deaths were reported for the cohort up until June 2018. Of individuals classified as low risk, 74 (2.6%) were identified as having died of CVD. Nine (7.7%) individuals classified at 10-20% risk and eight (15.9%) classified at >20% were identified as dying of CVD.

Conclusions: This study shows that there is a low risk profile of CVD in this slum population in Nairobi, Kenya in comparison to results from application of multivariable risk prediction tools in other LMIC populations. This has implications for health service planning in these contexts.

For peer review only - http://bmjopen.bmj.com/site/about/guidelines.xhtml

Strengths and limitations

- To the best of our knowledge this is the first study to apply a multivariable risk prediction tool to a population in a slum or informal settlement.
- We were able to identify CVD deaths of participants occurring in the slum during the 10 years after risk prediction.
- We were unable to exclude individuals with previous myocardial infarction as information was not available from the survey.
- Applying the risk score chart to cross-sectional population data may have underestimated the total CVD risk, as data that are required for thorough evaluation of total risk (such as family history) was absent.

peer review only

For peer review only - http://bmjopen.bmj.com/site/about/guidelines.xhtml

Introduction

Non-communicable diseases (NCDs) are the leading cause of death globally and have become the leading cause of death in low- and middle-income countries (LMICs).[1] Cardiovascular disease (CVD) is a key player in this epidemic, accounting for most NCD deaths, and studies of CVD in urban areas of LMICs have suggested that risk is growing.[2-4]

A large proportion of the world's urban population live in slums- neighbourhoods that are often informal, with poor housing and inadequate services.[5] There are conflicting views regarding CVD as a major public health problem for the urban poor, including those living in slums. An overview of health in slums found no synthesised evidence on CVD prevalence or the prevalence of CVD risk factors, while primary studies indicated that some CVD risk factors appear to be less prevalent among those living in slums than in their non-slum urban counterparts.[5] However, other primary studies carried out in urban LMICs have indicated that CVD risk is inversely associated with socio-economic status, or that there is no strong socio-economic gradient, which would suggest that those living in slums had at least equivalent or higher risk than other urban residents.[6, 7]

Conventionally, CVD risk prediction focused on the presence of certain individual risk factors (e.g. elevated blood pressure or serum cholesterol), however the recognition of the multifactorial aetiology of CVD has led to a drastic shift away from the single risk factor approach toward a multivariable risk prediction approach. Taking into account the co-existence of multiple risk factors to determine CVD risk has been supported by much research that clearly demonstrates that the risk of a CVD event can differ among individuals with the same high levels of single risk factors due to the presence or absence of other risk factors.[8-10] Furthermore, studies have shown that identifying individuals at high CVD risk by adopting a total CVD risk assessment approach is more cost-effective method of CVD prevention especially in low resource settings.[11, 12] Determining total CVD risk requires risk prediction tools. The World Health Organisation/International Society of Hypertension (WHO/ISH) developed risk score prediction charts for different WHO subregions for the purposes of enabling clinicians to quickly assess total CVD risk in their patients, but also allow for risk stratification of a population in a simple manner.[13]

However, there are no existing studies that have assessed multivariable risk prediction of CVD in a slum population. Therefore, the primary aim of this study is to apply the World Health Organisation/International Society of Hypertension (WHO/ISH) risk prediction tool to a slum population in Nairobi, Kenya. In addition, we were able to assess the number of cardiovascular-related deaths occurring within the slum (but not non-fatal events, or fatal events occurring elsewhere) reported within 10 years of application of the tool, giving us

For peer review only - http://bmjopen.bmj.com/site/about/guidelines.xhtml

some idea about the utility of the WHO/ISH tool in this population, and about the burden of CVD within the slum setting. These findings will inform plans for health service delivery in the context of urban poor settings.

Methods

Study Population

This study utilizes data from a cross-sectional survey conducted by the African Population and Health Research Center (APHRC) within the Nairobi Urban Health Demographic Surveillance Site (NUHDSS) population between May 2008 and April 2009. The NUHDSS was set up to examine the long-term social, economic and health effects of two slum communities within the city of Nairobi, Korogocho and Viwandani.[14] This population-based survey utilized the sampling frame from the NUHDSS and a stratified, sampling strategy based on the WHO STEPwise protocol with a target of 250 respondents in each of the following strata: sex, age group (18-24, 25-30, 31-40, 41-50, 51-60, and 60+), and slum of residence (Korogocho and Viwandani). Data were collected from a total of 5,470 individuals aged 18 years and above. Further details on the sampling frame and data collection methods are published elsewhere.[15] Men and women aged over the age of 40 years with complete data for variables needed for the WHO/ISH risk prediction tool were eligible to take part in this secondary data analysis.

Ethics

Operations of the NUHDSS are approved by the Government of Kenya, and the ethical responsibilities for its operations overseen by the Kenya Medical Research Institute (KEMRI). The CVD study was approved by the Scientific and Ethics Review unit of KEMRI (SERU NON-SSC 339). Participants were made aware that their participation in the study was voluntary, and individual informed consent was sought from all participants before their involvement in the study.

Study tool

In order to assess the 10-year risk of fatal & non-fatal cardiovascular disease (namely myocardial infarction or stroke) for each participant in our sample, we used the World Health Organisation/International Society of Hypertension (WHO/ISH) risk prediction charts for Africa sub-region (AFR E).[13] The charts are designed for those over 40 and those who do not have established coronary heart disease (CHD), stroke or other atherosclerotic disease. Therefore, our study sample excluded those <40 years of age and those with stroke. We

For peer review only - http://bmjopen.bmj.com/site/about/guidelines.xhtml

were unable to identify and exclude those who had established CHD or other atherosclerotic disease as the information was not available from the survey.

The chart requires data on sex, age, systolic blood pressure, smoking status and presence or absence of diabetes mellitus. If total serum cholesterol is available, this is used in CVD risk prediction, however there is also an algorithm for use where no total serum cholesterol record is available, which we used to calculate risk for the study participants with missing cholesterol data. Studies have demonstrated high correlation between laboratory-based scores and non-laboratory based scores for men and women.[16, 17]

Following guidelines for using the WHO/ISH risk prediction tool variables were constructed as follows: smokers were considered as those who were current smokers at assessment or those who quit smoking within the last year before the assessment; presence of diabetes was defined as someone taking insulin or oral hypoglycaemic drugs or having a study-measured fasting glucose >7.0 mmol/l; systolic blood pressure was the average of three readings on the day of survey while study member was seated using OMRON M6 blood pressure machine; total cholesterol (mmol/l) was measured by taking capillary blood from fingertips using the ACCUCHECK GCT monitors and test strips. The predicted risk falls into categories including: from <10% (low), 10- <20% (moderate), 20- <30% (high), 30- <40% (very high) and 40% or more (highest).[18]

In addition, practice points accompany the WHO risk prediction charts and state that CVD risk may be elevated over that specified by the charts when certain factors are present. We were able to obtain the following CVD enhancing risk factors for our study members: raised triglycerides (>2.0 mmol/l), whether on hypertensive medication and presence of obesity defined according to BMI (weight in kg divided by the square of height in cm). The prevalence of these CVD risk elevating factors were tabulated by the different risk categories.

Identifying cardiovascular related deaths within 10 years of application of the tool

To assess the cardiovascular related deaths among the participants of this study, verbal autopsy data present for all deaths recorded between 2008 and June 2018 was obtained from APHRC. A record linkage was undertaken between the cross-sectional survey and the verbal autopsy data using a unique identifier present in both data sources.

Verbal autopsy interviews are conducted by experienced field interviewers with a "credible respondent", usually a family member following identification of deaths during regular Demographic Surveillance Site (DSS) data collection.[19] A standardised questionnaire

For peer review only - http://bmjopen.bmj.com/site/about/guidelines.xhtml

developed in conjunction with other International Network of Demographic Evaluation of Populations and Their Health (INDEPTH) sites is used and consists of open and closed questions focusing on events leading to the death and specific clinical signs and symptoms that the deceased had prior to their death. After several visits to a household, if no "credible respondent" is identified, verbal autopsy is coded as missing, and no cause of death is recorded.

Cause of death is then generated using InterVA-4 software, which uses probabilistic models based on Bayes' theorem to interpret symptom and signs data from verbal autopsy questionnaires and determine possible causes of death. Detailed information of the InterVA model and how it was developed have been described in previous studies.[20-22] Those with a cardiovascular code recorded under the variable "first broad cause of death" was used to define cardiovascular-related death in this study. CV death included ischaemic heart disease, cerebrovascular disease, hypertensive diseases, pulmonary heart disease and diseases of pulmonary circulation, and diseases of arteries, arterioles and capillaries. We additionally examined deaths reported due to diabetes mellitus because someone who had diabetes at the time of death may have died from a cardiovascular outcome, but credible family members may only have discussed the condition they were suffering prior to death.

Please note that the deaths recorded in this study were deaths only identified during the regular data collection rounds by the DSS team, it may be that more deaths occurred among participants of the original cross-sectional survey that were not identified (for example, the participant had moved before the death took place) and non-fatal CVD events were not captured at all. We were not able to link individuals in this dataset to know if they were still resident in the NUHDSS in June 2018- but a larger study sample, including this one, identified just 53/4290 (1.2%) had exited the NUHDSS between 2008 and 2018 (Frederick Wekesah- personal communication). The published rate of out migration between 2003 and 2012 was 22.5%.[14] For these reasons we can't be sure how well the tool predicted CVD events in this population but include these figures to add to knowledge about the burden of disease and give some indication of the tool's performance.

Patients and public were not involved in this study.

Statistical Analyses

Data analysis was conducted in STATA, version 14.0 (StataCorp. LP, College Station, USA). Percentages were calculated for categorical variables. Sampling weights were applied where noted.

For peer review only - http://bmjopen.bmj.com/site/about/guidelines.xhtml

Results

Description of sample

Of the 5470 survey participants, 2,316 were excluded due to age (<40 years). Of the remaining 3,154 participants aged over 40, 10 were excluded due to having a stroke. 81 (2.6%) were excluded due to incomplete data for the variables required of the WHO/ISH risk prediction tool (missing data for smoking status (n=2) and for blood pressure (n=81)).

Characteristics of the 3063 participants included in the final sample are represented in Table 1. The majority of participants included in the analyses were male (57.6%) and were between 40-60 years old, 88% of participants included in the analyses were non-smokers, 3% had diabetes and approximately 24% had blood pressure ≥140 mmHg.

Table 1: Characteristics of study participants

	Total (n= 3063)	Men (n=1765)	Women (n=1298)
**Age in years (n, %)**			
40-49	1168 (38.13)	595 (33.71)	573 (44.14)
50-59	1169 (38.17)	770 (43.63)	399 (30.74)
60-69	493 (16.10)	294 (16.66)	199 (15.33)
≥70	233 (7.61)	106 (6.01)	127 (9.78)
**BMI category (n, %))**			
<30 kg/m^2	2714 (89.39)	1700 (96.98)	1014 (79.03)
≥30 kg/m^2	322 (10.61)	53 (3.02)	269 (20.97)
**Smoking status (n, %)**			
Current	375 (12.24)	364 (20.62)	11 (0.85)
Non-smoker	2688 (87.76)	1401(79.38)	1287 (99.15)
**Blood pressure (n, %)**			
<140	2312 (75.48)	1382 (78.30)	939 (72.34)
140-159	441 (14.40)	237 (13.43)	204 (15.72)
160-179	185 (6.04)	101 (5.72)	84 (6.47)
180+	116 (3.79)	45 (2.55)	71 (5.47)
**Diabetes (n, %)**			
Absent	2976 (97.16)	1727 (97.85)	1249 (96.22)
Present	87 (2.84)	38 (2.15)	49 (3.78)
**Total Cholesterol (n, %)**			
<5	2220 (72.48)	1307 (74.05)	913 (70.33)
5-5.9	516 (16.84)	281 (15.92)	235 (18.10)

For peer review only - http://bmjopen.bmj.com/site/about/guidelines.xhtml

6-6.9	74 (2.42)	32 (1.81)	42 (3.24)
7-7.9	10 (0.33)	5 (0.28)	5 (0.39)
8+	2 (0.07)	1 (0.06)	1 (0.08)
Cholesterol missing	241(7.87)	139 (7.88)	102 (7.86)

Table 2: Proportion of population % at different level of CVD risk & Prevalence of CVD risk enhancing factors

10-year CVD risk	<10% Low	10 to <20% Moderate	20% to < 30% High risk	30% to <40% Very High risk	≥40% Highest risk
**Total**					
CVD risk-prediction from WHO/ISH chart alone (n, %)	2895 (94.5)	117 (3.8)	31 (1.0)	14 (0.5)	6 (0.2)
CVD risk-prediction from WHO/ISH chart alone (%) (weighted)	(96.3)	(2.5)	(0.8)	(0.3)	(0.1)
One or more CVD enhancing factors (n, % of risk category)	932 (32.2)	62 (53.0)	20 (64.5)	3 (21.4)	5 (83.3)
Obesity (BMI ≥ 30) (n, % of risk category [a])	292 (10.2)	21 (18.3)	9 (29.0)	0 (0)	0 (0)
On anti-hypertensive medication (n, % of risk category)	75 (2.6)	20 (17.1)	6 (19.4)	1 (7.1)	2 (33.3)
High Triglycerides (n, % risk category [b])	714 (27.6)	46 (42.6)	12 (44.4)	3 (25.0)	4 (80.0)
**Male**					
CVD risk-prediction from WHO/ISH chart alone (n, %)	1679 (95.1)	59 (3.3)	12 (0.7)	11 (0.6)	4 (0.2)
CVD risk-prediction from WHO/ISH chart alone (%) (weighted)	(95.2)	(3.5)	(1.1)	(0.2)	(0.1)
One or more CVD enhancing factors (n, % of risk category)	443 (26.4)	21 (35.6)	6 (50.0)	2 (18.1)	3 (75.0)

For peer review only - http://bmjopen.bmj.com/site/about/guidelines.xhtml

For peer review only

Obesity (BMI ≥ 30) (n, % of risk category [a])	49 (2.9)	2 (3.4)	2 (16.7)	0 (0.0)	0 (0.0)
On anti-hypertensive medication (n, % of risk category)	22 (1.3)	6 (10.2)	1 (8.3)	0 (0.0)	0 (0.0)
High Triglycerides (n, % risk category [b])	400 (26.6)	16 (30.2)	4 (44.4)	2 (22.2)	3 (75.0)
**Female**					
CVD risk-prediction from WHO/ISH chart alone (n, %)	1216 (93.7)	58 (4.5)	19 (1.5)	3 (0.2)	2 (0.2)
CVD risk-prediction from WHO/ISH chart alone (%) (weighted)	(96.8)	(2.1)	(0.7)	(0.3)	(0.2)
One or more CVD enhancing factors (n, % of risk category)	489 (40.2)	41 (70.7)	14 (73.7)	1 (33.3)	2 (100)
Obesity (BMI ≥ 30) (n, % of risk category [a])	243 (20.2)	19 (33.9)	7 (36.8)	0 (0.0)	0 (0.0)
On anti-hypertensive medication (n, % of risk category)	53 (4.4)	14 (24.1)	5 (26.3)	1 (33.3)	2 (100)
High Triglycerides (n, % risk category [b])	314 (28.9)	30 (54.5)	8 (44.4)	1 (33.3)	1 (100)

[a] *Realistic BMI values only available for 3036 participants of our total sample size of 3063*

[b] *High Triglycerides were recorded in 2739 participants of our total sample size of 3063*

For peer review only

The majority of participants in this study sample had low (<10%) total 10-year CVD risk (2895, 94.5%) [Table 2]. That is, they had less than 10% predicted chance of a fatal or non-fatal CVD event over the following 10 years. When CVD-risk enhancing factors were taken into account, 1963 participants (64.1%) had low (<10%) total 10-year CVD risk with no additional risk enhancing factors. In the weighted analysis, the percentage of people in the <10% risk group was 96.3% with the reduced risk profile due to correction of the over-sampling of older age groups. After applying the CVD-enhancing factors, the percentage of people in the <10% risk group reduced to 63.7% in the weighted analysis.

WHO also states risk from prediction charts alone can underestimate the risk in those with high blood pressure (≥160/100) or blood cholesterol ≥8. In our sample, 9.83% had raised

blood pressure but only 0.07% of those who had a cholesterol blood test were raised above the specified level. Of those at <10% risk group, 131 participants had raised blood pressure above or equal to 160/100 and of those in this category that had a blood cholesterol test only two participants had a blood cholesterol ≥8.

Table 3: Total Deaths and deaths due to cardiovascular disease recorded up to June 2018 at different levels of predicted CVD risk (based on chart alone)

10-year CVD risk	<10% Low	10-20% Moderate	20-30% High risk	30-40% Very High risk	≥40% Highest risk
**Total**					
Deaths recorded (n, %)	336 (11.6)	25 (21.4)	11 (35.5)	7 (50.0)	1 (16.7)
Broad 1st cause of death: Cardiovascular (n, %)	74 (2.6)	9 (7.7)	5 (16.1)	2 (14.3)	1 (16.7)
Broad 1st cause of death: Diabetes Mellitus (n, %)	8 (0.3)	0 (0.0)	0 (0.0)	0 (0.0)	0 (0.0)
Indeterminate cause (n, %)	30 (1.0)	4 (3.4)	0 (0.0)	0 (0.0)	0 (0.0)
VA not done (n, %)	33 (1.1)	1 (0.9)	0 (0.0)	0 (0.0)	0 (0.0)
**Male**					
Deaths recorded (n, %)	199 (11.9)	10 (16.9)	5 (41.7)	4 (36.4)	0 (0.0)
Broad 1st cause of death: Cardiovascular (n, %)	35 (2.1)	3 (5.1)	2 (16.7)	1 (9.1)	0 (0.0)
Broad 1st cause of death: Diabetes Mellitus (n, %)	3 (0.2)	0 (0.0)	0 (0.0)	0 (0.0)	0 (0.0)
Indeterminate cause (n, %)	16 (1.0)	1 (1.7)	0 (0.0)	0 (0.0)	0 (0.0)
VA not done (n, %)	22 (1.3)	1 (1.7)	0 (0.0)	0 (0.0)	0 (0.0)

For peer review only - http://bmjopen.bmj.com/site/about/guidelines.xhtml

For peer review only

Female					
Deaths recorded (n, %)	137 (11.3)	15 (25.9)	6 (31.6)	3 (100)	1 (50.0)
Broad 1st cause of death: Cardiovascular (n, %)	36 (3.1)	6 (10.3)	3 (15.8)	1 (33.3)	1 (50.0)
Broad 1st cause of death: Diabetes Mellitus (n, %)	5 (0.4)	0 (0.0)	0 (0.0)	0 (0.0)	0 (0.0)
Indeterminate cause (n, %)	141 (11.6)	3 (5.2)	0 (0.0)	0 (0.0)	0 (0.0)
VA not done (n, %)	11 (0.9)	0 (0.0)	0 (0.0)	0 (0.0)	0 (0.0)

Following record linkage of verbal autopsy database and the respondents of the cross-sectional survey, 466 records were matched i.e. 466 of the original cohort had a death reported up to June 2018 among the participants from the cross-sectional survey (n=5,470). Among the 3063 participants included in this study, 410 deaths were recorded with 91 deaths specifically related to CVD (3% of the study population), while 34 were classified as indeterminate and in 34 further cases, a verbal autopsy was not performed. Cardiovascular related cause of death was assigned to 74 (2.6%) of individuals classified as low risk (<10% predicted chance of a fatal or non-fatal CVD event). Nine (7.7%) of individuals classified at 10-20% risk of a fatal or non-fatal CVD event were determined to have died from CVD, and 8 (15.9%) of those at high risk (≥20%) [Table 3]. Out of 336 deaths in <10% group, 87 individuals who had died had one or more CVD-enhancing factors, 18 of these deaths were due to CVD risk and two deaths were due to diabetes.

Discussion

Of the 3063 study members aged over 40, the majority (94.5%) were predicted to have a less than 10% chance of experiencing a cardiovascular event over the next 10 years and just 1.7% having a "high" CVD risk (≥20%). When weighted to be representative of all adults aged over 40 living in the slum 96.3% were predicted to fall in the lowest risk group and just 1.2% have a "high" CVD risk (≥20%). This is a low risk population profile in comparison to results from application of the multivariable risk prediction tools in other populations. Studies conducted specifically among urban dwellers in LMIC countries such as Malaysia [23] and Sri

For peer review only - http://bmjopen.bmj.com/site/about/guidelines.xhtml

Lanka [24] have found 20.5% and 8.2% individuals were at high risk (≥20%) of having a future CVD event, respectively. Furthermore, the proportion of individuals in our study shown to be at low risk of a CVD event over 10 years (<10% risk) was higher than that of studies who used the WHO/ISH risk prediction charts carried out in rural Nepal (86.4%),[25] rural South India (83%) [26] and rural Bangladesh (81.3%).[27] Mendis et al. reported total 10-year CVD risk in defined geographical areas of seven countries including both urban and rural populations, but only two countries had a higher percentage of individuals classified as low risk in comparison to our study (lower: Iran (93.9%), Cuba (89.7%), Nigeria (86.0%), Georgia (83.1%), Pakistan (79.2%); similar: China (96.1%) and Sri Lanka (94.9%)).[28] However, it is important to note, the proportion of individuals estimated to have low (<10%) total CVD risk substantially decreased in our study, when risk-elevating factors stated in practice points accompanying WHO/ISH charts (raised triglycerides, obesity and anti-hypertensive medication) were added to the CVD risk assessment of the population.

CVD deaths occurring in our study population within the slum, reflected risk-categories assigned by the WHO/ISH tool (bearing in mind that additional deaths may have occurred in study members outside the slum and that non-fatal events were not recorded). Taken as a whole, it appears from this data that health services geared towards CVD treatment may be less of a priority in slum settings in Kenya, or potentially in sub-Saharan Africa, than in the wider urban areas of LMIC cities. An important reason for this may be the age-structure of the slum population, which is very young. However, given the large percentage of CVD-enhancing factors in this population, it could be that the future burden (when this population gets older) will be significant. The signal here could be that CVD prevention is more of a priority here than treatment.

Certain limitations of this work need to be considered when interpreting the findings. First, we were unable to exclude individuals with previous myocardial infarction as information was not available from the survey. However, if we failed to identify significant numbers with a previous MI, the remaining population (once these individuals had been excluded) would have likely had an even more extremely low risk profile for CVD. Second, applying the risk score chart to cross-sectional population data may have underestimated the total CVD risk, as data that are required for thorough evaluation of total risk such as family history or even history of relevant current diseases (the obvious example being myocardial infarction) and treatments, were not present in the data. Thirdly, there are some deviations in our methods from the instructions of how the WHO/ISH charts should be used: systolic blood pressure was measured three times on one day, rather than twice at two different time points, which could increase the risk that some of the participants experienced white-coat hypertension; we

For peer review only - http://bmjopen.bmj.com/site/about/guidelines.xhtml

defined someone as having diabetes if they were taking insulin or oral hypoglycaemic drugs or if their fasting plasma glucose concentration was about 7.0mmol/l on one occasion (not on two separate occasions as recommended). Finally, where we used cholesterol readings- these were also from one time point, rather than two as recommended.

Finally, it is a regret that we don't have data on all possible fatal CVD events (for example in those who have moved from the study site and are therefore not followed up in the NUHDSS) or non-fatal events that have occurred in the 10 year since the risk data was collected in order to validate the WHO/ISH tool in this setting.

Despite these limitations, our study uses rare data to provide a good estimate of total 10-year CVD risk among a marginalised population in an urban poor setting in sub-Saharan Africa. To the best of our knowledge this is the first study to apply a multivariable risk prediction tool to a population in a slum or informal settlement and to assess the number of cardiovascular related deaths within 10 years of application of the tool. This study shows that there is a low risk profile of CVD in this slum population in Nairobi, Kenya and that the WHO/ISH tool does differentiate groups at increasing risk of CVD mortality. This has implications for planning of health service delivery in slums.

Conflict of Interest: None to declare

Funding: OO and CK are supported by the National Institute for Health Research (NIHR) Research Unit on Improving Health in Slums. The original data on which the study is based was collected as part of research funded by the Wellcome Trust UK- Grant Number WT092775MA.This research did not receive any specific grant from funding agencies in the public, commercial, or not-for-profit sectors. This paper presented independent research and the views expressed are those of the authors and not necessarily those of the NHS, the HIGR or the Department of Health.

Contributors: OO conceived the study idea. OO, AV and FW contributed to the analysis plan. AV conducted the analyses. FW and CK provided advice on using the data. AV and OO wrote the first draft of the manuscript. All authors contributed to the final manuscript.

Competing interests: None declared.

Data sharing statement: No additional data available

For peer review only

REFERENCES

1 Alwan A, Armstrong T, Bettcher D, et al. Global status report on noncommunicable diseases 2010: description of the global burden of NCDs, their risk factors and determinants. *World Health Organization* 2011.

2 Yusuf S, Rangarajan S, Teo K, et al. Cardiovascular risk and events in 17 low-, middle-, and high-income countries. *N Engl J Med* 2014;371:818-27.

3 Gaziano TA, Bitton A, Anand S, et al. Growing epidemic of coronary heart disease in low- and middle-income countries. *Curr Probl Cardiol* 2010;35:72-115.

4 Teo KK, Dokainish H. The emerging epidemic of cardiovascular risk factors and atherosclerotic disease in developing countries. *Can J Cardiol* 2017;33:358-65.

5 Ezeh A, Oyebode O, Satterthwaite D, et al. The history, geography, and sociology of slums and the health problems of people who live in slums. *The lancet* 2017;389:547-58.

6 Marins VM, Almeida RM, Pereira RA, et al. The association between socioeconomic indicators and cardiovascular disease risk factors in Rio de Janeiro, Brazil. *J Biosoc Sci* 2007;39:221-9.

7 Vellakkal S, Subramanian S, Millett C, et al. Socioeconomic inequalities in non-communicable diseases prevalence in India: disparities between self-reported diagnoses and standardized measures. *PloS one* 2013;8:e68219.

8 Volpe M, Alderman MH, Furberg CD, et al. *Beyond hypertension: Toward guidelines for cardiovascular risk reduction* 2004.

9 Andersson OK, Almgren T, Persson B, et al. Survival in treated hypertension: follow up study after two decades. *BMJ* 1998;317:167-71.

10 Tocci G, Valenti V, Sciarretta S, et al. Multivariate risk assessment and risk score cards in hypertension. *Vasc Health Risk Manag* 2007;3:313-20.

11 Ndindjock R, Gedeon J, Mendis S, et al. Potential impact of single-risk-factor versus total risk management for the prevention of cardiovascular events in Seychelles. *Bull World Health Organ* 2011;89:286-95.

12 Mendis S, Lindholm LH, Anderson SG, et al. Total cardiovascular risk approach to improve efficiency of cardiovascular prevention in resource constrain settings. *J Clin Epidemiol* 2011;64:1451-62.

13 . WHO/ISH Risk prediction charts for 14 WHO epidemiological sub-regions. 2007. Available from: http://ish-world.com/downloads/activities/colour_charts_24_Aug_07.pdf (accessed 18 Jun 2019).

14 Beguy D, Elung'ata P, Mberu B, et al. Health & demographic surveillance system profile: the Nairobi urban health and demographic surveillance system (NUHDSS). *Int J Epidemiol* 2015;44:462-71.

For peer review only - http://bmjopen.bmj.com/site/about/guidelines.xhtml

15 Van de Vijver, Steven JM, Oti SO, Agyemang C, et al. Prevalence, awareness, treatment and control of hypertension among slum dwellers in Nairobi, Kenya. *J Hypertens* 2013;31:1018-24.

16 Pandya A, Weinstein MC, Gaziano TA. A comparative assessment of non-laboratory-based versus commonly used laboratory-based cardiovascular disease risk scores in the NHANES III population. *PloS one* 2011;6:e20416.

17 Gaziano TA, Young CR, Fitzmaurice G, et al. Laboratory-based versus non-laboratory-based method for assessment of cardiovascular disease risk: the NHANES I Follow-up Study cohort. *The Lancet* 2008;371:923-31.

18 Organisation mondiale de la santé. Prevention of cardiovascular disease: guidelines for assessment and management of cardiovascular risk: World Health Organization 2007.

19 Oti SO, Kyobutungi C. Verbal autopsy interpretation: a comparative analysis of the InterVA model versus physician review in determining causes of death in the Nairobi DSS. *Population health metrics* 2010;8:21.

20 Byass P, Huong DL, Van Minh H. A probabilistic approach to interpreting verbal autopsies: methodology and preliminary validation in Vietnam. *Scand J Public Health* 2003;31:32-7.

21 Oti SO, Kyobutungi C. Verbal autopsy interpretation: a comparative analysis of the InterVA model versus physician review in determining causes of death in the Nairobi DSS. *Population health metrics* 2010;8:21.

22 Byass P, Fottrell E, Huong DL, et al. Refining a probabilistic model for interpreting verbal autopsy data. *Scand J Public Health* 2006;34:26-31.

23 Su TT, Amiri M, Mohd Hairi F, et al. Prediction of cardiovascular disease risk among low-income urban dwellers in metropolitan Kuala Lumpur, Malaysia. *BioMed research international* 2015;2015.

24 Ranawaka U, Wijekoon C, Pathmeswaran A, et al. Risk estimates of cardiovascular diseases in a Sri Lankan community. *Ceylon Med J.* 2016;61(1):11-7

25 Khanal MK, Ahmed MM, Moniruzzaman M, et al. Total cardiovascular risk for next 10 years among rural population of Nepal using WHO/ISH risk prediction chart. *BMC research notes* 2017;10:120.

26 Ghorpade AG, Shrivastava SR, Kar SS, et al. Estimation of the cardiovascular risk using World Health Organization/International Society of Hypertension (WHO/ISH) risk prediction charts in a rural population of South India. *International journal of health policy and management* 2015;4:531.

27 Ahmed M, Moniruzzaman M, Chowdhury S, et al. Cardiovascular Risk Assessment Among Urban Population of Bangladesh Using WHO/ISH Risk Prediction Chart. *Int J Epidemiol* 2015;44:i202.

28 Mendis S, Lindholm LH, Anderson SG, et al. Total cardiovascular risk approach to improve efficiency of cardiovascular prevention in resource constrain settings. *J Clin Epidemiol* 2011;64:1451-62.

For peer review only - http://bmjopen.bmj.com/site/about/guidelines.xhtml

For peer review only

STROBE Statement—Checklist of items that should be included in reports of ***cross-sectional studies***

	Item No	Recommendation	Reported on Page no.
**Title and abstract**	1	(*a*) Indicate the study's design with a commonly used term in the title or the abstract	1
		(*b*) Provide in the abstract an informative and balanced summary of what was done and what was found	2
**Introduction**			
Background/rationale	2	Explain the scientific background and rationale for the investigation being reported	4
Objectives	3	State specific objectives, including any prespecified hypotheses	4
**Methods**			
Study design	4	Present key elements of study design early in the paper	5-7
Setting	5	Describe the setting, locations, and relevant dates, including periods of recruitment, exposure, follow-up, and data collection	5-7
Participants	6	(*a*) Give the eligibility criteria, and the sources and methods of selection of participants	5
Variables	7	Clearly define all outcomes, exposures, predictors, potential confounders, and effect modifiers. Give diagnostic criteria, if applicable	5-7
Data sources/ measurement	8*	For each variable of interest, give sources of data and details of methods of assessment (measurement). Describe comparability of assessment methods if there is more than one group	5-7
Bias	9	Describe any efforts to address potential sources of bias	N/A
Study size	10	Explain how the study size was arrived at	8
Quantitative variables	11	Explain how quantitative variables were handled in the analyses. If applicable, describe which groupings were chosen and why	6-7
Statistical methods	12	(*a*) Describe all statistical methods, including those used to control for confounding	7
		(*b*) Describe any methods used to examine subgroups and interactions	N/A
		(*c*) Explain how missing data were addressed	6
		(*d*) If applicable, describe analytical methods taking account of sampling strategy	5, 7
		(*e*) Describe any sensitivity analyses	N/A
**Results**			
Participants	13*	(a) Report numbers of individuals at each stage of study—eg numbers potentially eligible, examined for eligibility, confirmed eligible, included in the study, completing follow-up, and analysed	8
		(b) Give reasons for non-participation at each stage	8
		(c) Consider use of a flow diagram	N/A
Descriptive data	14*	(a) Give characteristics of study participants (eg demographic, clinical, social) and information on exposures and potential confounders	8-9
		(b) Indicate number of participants with missing data for each variable of interest	8-10
Outcome data	15*	Report numbers of outcome events or summary measures	9-12
Main results	16	(*a*) Give unadjusted estimates and, if applicable, confounder-adjusted estimates and their precision (eg, 95% confidence interval). Make clear	N/A

For peer review only

		which confounders were adjusted for and why they were included	
		(*b*) Report category boundaries when continuous variables were categorized	N/A
		(*c*) If relevant, consider translating estimates of relative risk into absolute risk for a meaningful time period	N/A
Other analyses	17	Report other analyses done—eg analyses of subgroups and interactions, and sensitivity analyses	N/A
**Discussion**			
Key results	18	Summarise key results with reference to study objectives	12-13
Limitations	19	Discuss limitations of the study, taking into account sources of potential bias or imprecision. Discuss both direction and magnitude of any potential bias	13-14
Interpretation	20	Give a cautious overall interpretation of results considering objectives, limitations, multiplicity of analyses, results from similar studies, and other relevant evidence	14
Generalisability	21	Discuss the generalisability (external validity) of the study results	14
**Other information**			
Funding	22	Give the source of funding and the role of the funders for the present study and, if applicable, for the original study on which the present article is based	14

*Give information separately for exposed and unexposed groups.

Note: An Explanation and Elaboration article discusses each checklist item and gives methodological background and published examples of transparent reporting. The STROBE checklist is best used in conjunction with this article (freely available on the Web sites of PLoS Medicine at http://www.plosmedicine.org/, Annals of Internal Medicine at http://www.annals.org/, and Epidemiology at http://www.epidem.com/). Information on the STROBE Initiative is available at www.strobe-statement.org.

For peer review only

For peer review only - http://bmjopen.bmj.com/site/about/guidelines.xhtml
